# The Role of AGT, AMPD1, HIF1α, IL-6 Gene Polymorphisms in the Athletes’ Power Status: A Meta-Analysis

**DOI:** 10.5114/jhk/169262

**Published:** 2023-09-05

**Authors:** Gokhan Ipekoglu, Tugba Cetin, Necdet Apaydin, Tugce Calcali, Ebru Senel

**Affiliations:** 1 Faculty of Sport Science, Ordu University, Ordu, Turkey.; 2 School of Physical Education and Sports, Karabuk University, Karabuk, Turkey.; 3 Faculty of Sport Sciences, Giresun University, Giresun, Turkey.

**Keywords:** sport genetics, gene, meta-analysis, athletic performance

## Abstract

This meta-analysis was designed to investigate the relationship between genetic polymorphisms (AGT rs699, AMPD1 rs17602729, HIF1α rs11549465, IL-6 rs1800795) and power athletes’ status. Only case-control studies were included in the meta-analysis. A systematic search of the PubMed and Web of Science databases was performed to identify relevant studies and 23 studies met the inclusion criteria for the meta-analysis. The data from the included studies were pooled and analyzed using a random effects or fix effects model. The effect size was calculated as the odds ratio or a risk ratio with 95% confidence intervals. The results showed that certain genetic polymorphisms, AGT rs699 Thr allele, HIF1A rs11549465 Ser allele and AMPD1 rs17602729 C allele, were significantly more prevalent in power athletes (p < 0.05). When examining the genotype frequency distribution of AGT rs699 and AMPD1 rs17602729, significant differences were found in both the dominant and recessive models (p < 0.05). The results indicate that these gene polymorphisms play a role in power athlete status, however, new and more comprehensive studies are needed to confirm these results.

## Introduction

With the increase of molecular research in sports, genetic factors related to power and endurance performance have been discovered (Ahmetov and Fedotovskaya, 2012; Pitsiladis et al., 2013). Sports genetics is a scientific discipline based on the genetic organization and function of professional athletes (Ahmetov and Fedotovskaya, 2015). Genetic research in the field of sports and exercise encompasses a broad range of investigations beyond just DNA polymorphisms and gene associations. It also includes the study of various genetic factors, such as gene expression, epigenetics, and protein function, which collectively contribute to understanding athletic performance, training adaptation, and musculoskeletal injuries resulting from exercise (Ipekoglu et al., 2022; Maciejewska-Skrendo et al., 2021). In power sports, muscle strength is an important characteristic for athletes to achieve peak performance. The genetic structure of athletes can influence muscle fiber characteristics such as the type, size, and the ratio of different fiber types, which can inform the identification of favourable genotypes associated with power sports (Weyerstraß et al., 2018). Recently, the most popular genes studied in relation to power athlete status are *AGT* (Angiotensin), *AMPD1* (adenosine monophosphate deaminase), *HIF1α* (Hypoxia-inducible factor 1), and *IL-6* (Interleukin-6).

The angiotensinogen (*AGT*) gene encodes a protein that plays a key role in the renin-angiotensin-aldosterone system, which regulates blood pressure and fluid balance in the body. AGT is located on chromosome 1q42 (Corvol and Jeunemaitre, 1997). The AGT gene has several single nucleotide polymorphisms (SNPs) that have been studied in relation to athletic performance and power athlete status. Rauramaa et al. (2002) have found that the M235T polymorphism is associated with increased angiotensinogen levels and may influence blood pressure and cardiovascular responses to exercise. Other studies have suggested that the M235T polymorphism may be related to muscle strength and power athlete status, with the C allele being associated with improved athletic performance in power-based sports (Gomez-Gallego et al., 2009; Zarebska et al., 2013).

Adenosine monophosphate deaminase (AMPD1) plays a crucial role in regulating muscle-energy metabolism during exercise, and is also referred to as myoadenylate deaminase. It is predominantly expressed in all types of skeletal muscle fibers and is encoded by a gene located on chromosome 1 (1p13). In particular, AMPD1 is mostly found in fast-twitch (type II) muscle fibers. It has been suggested that variations in AMPD1 C34T (rs17602729) gene expression may contribute to differences in enzyme activity across different groups of muscle fiber types. During intense exercise, ATP may be depleted by 50%, and it is thought that individuals with low AMPD activity may not be able to effectively perform short-term high-intensity exercise (Fedotovskaya et al., 2013; Rubio et al., 2005). Low AMPD activity has been observed in individuals with the TT genotype, intermediate activity in those with the CT genotype, and normal activity in those with the CC genotype. The AMPD1 C allele may help athletes achieve elite status in power-based sports (Dias et al., 2007; Faul et al., 2007; Ginevičiene et al., 2014).

The HIF1A gene, responsible for encoding the α subunit of HIF-1 protein, was found to exhibit the Pro582Ser polymorphism (rs11549465). This polymorphism entails the substitution of proline (Pro) with serine (Ser) at amino acid position 582 within exon 12 (Ahmetov and Fedotovskaya, 2015). The hypoxia-inducible transcription factor HIF1α plays an important role in the regulation of glucose, an anaerobic energy source, under low oxygen conditions (Gabbasov et al., 2013). HIF1a is stimulated by various factors. Oxygen deficiency observed in muscle tissue with high intensity of neuromuscular function is typical for power-based sports (Ciȩszczyk et al., 2011). The increased transcription activity of the Ser allele rather than the Pro allele of the Pro582Ser polymorphism identified in the HIF1α gene has been associated with increased stability of the HIF1α protein and therefore increased hypoxic resistance in cells (Tanimoto et al., 2003). Based on these data, it can be said that carriers of the Ser allele are more prone to power-demanding sports. A study found that the HIF1α Pro582Ser variant was significantly higher in weightlifters and wrestlers compared to the control group (Gabbasov et al., 2013).

Interleukin-6 (*IL-6*) is a cytokine with diverse biological functions that include regulating differentiation, proliferation, survival, and immune acute phase response in target cells. Although primarily produced by immune cells, it is also expressed in muscle cells and its secretion increases during muscle contraction (Febbraio and Pedersen, 2005). Skeletal muscle produces IL-6 to enhance substrate delivery and potentially aid in reducing inflammation after exercise (Petersen and Pedersen, 2005). Elevated plasma IL-6 levels during physical exercise are attributed to its release from muscles involved in metabolic processes. IL-6 is also known to play a role in glucose homeostasis regulation during exercise and contribute to hypertrophic muscle growth through satellite cells (Serrano et al., 2008). The IL-6 promoter gene (located at 7p21) contains the 174 C/G (rs1800795) polymorphism, which modulates transcriptional response. In the context of human performance, the presence of the C allele and the CC genotype has been linked to increased creatine kinase activity following eccentric exercise. There is a genetically determined difference in IL-6 response to stressful stimuli among individuals, with the C allele being significantly associated with lower plasma IL-6 levels (Ahmetov and Fedotovskaya, 2015). The GG genotype and the G allele have been observed to have a higher allele frequency in elite power athletes compared to endurance athletes and control individuals (Ruiz et al., 2010).

The aim of this meta-analysis was to synthesize the current evidence on the relationship between four genetic polymorphisms (*AGT* rs699 *, AMPD1* rs17602729 *, HIF1α* rs11549465 *, IL-6* rs1800795). By expanding the scope of genetic analysis, this study aimed to enhance our understanding of the role of genetics in the athletes’ strength status, acknowledging that the analysis of these four gene polymorphisms alone may provide only a limited perspective.

## Methods

### Selection of Studies

A systematic search of the PubMed and Web of Science databases was conducted to identify relevant studies on *AGT* rs699 *, AMPD1* rs17602729 *, HIF1α* rs11549465 *, IL-6* rs1800795 gene polymorphisms and power athlete status. The search included studies published from 2000 to 2022 and used the following keywords: “*AMPD1* polymorhism”, “*AGT* polymorhism”, “*HIF1α* polymorhism”, “*IL-6* polymorhism”, “genetic polymorphism”, in combination with “athlete” and “power”. The reference lists of relevant articles were also reviewed to identify additional studies. A total of 458 studies were initially identified, of which 23 met the inclusion criteria for the meta-analysis. The inclusion criteria were as follows: (1) the study examined the association between the *AMPD1, AGT, HIF1α, IL-6* gene polymorphism and power athlete status, (2) the study included a case and a control group, and (3) the study provided sufficient data to calculate an odds ratio or a risk ratio with 95% confidence intervals. The exclusion criteria were as follows: (1) the study was a review article, (2) the study was a case report, and (3) the study was not relevant to the research question.

### Data Extraction

Two independent reviewers extracted data from the included studies using a standardized form. The following information was collected: study characteristics such as the publication year, the country of origin, and the study design; study population characteristics such as age, sex, and ethnicity; the type of gene polymorphisms under investigation; and outcome measures such as power athlete status. Any discrepancies between the two reviewers were resolved through discussion and consensus.

### Eligibility Criteria

Athlete-control articles including *AMPD1, AGT, HIF1-α*, and *IL-6* gene polymorphisms associated with power sports were included in the study. On the other hand, meta-analyses that contained unclear data, did not include a control group, and those of which full text could not be accessed were excluded from this meta-analysis.

### Statistical Analysis

The data from the included studies were pooled using random effects or fix effects models due to the significant heterogeneity among the studies. The presence of heterogeneity was assessed using the Cochran Q test and the I2(%) statistic. The presence of publication bias was assessed using funnel plots and the Egger test. The effect size was calculated as the odds ratio or the risk ratio with 95% confidence intervals. Statistical analysis was performed using Jamovi2.3.

## Results

Table 1 shows the descriptive information of the author, year, race, related sport branch, studied gene names, total number of power athletes and control groups of the 23 studies included in the meta-analysis.

**Table 1:** Descriptive table of included studies.

Study	Year	Race	Related Sports	Name Genes (rs number)	Total Athletes	Total Controls
Ahmetov et al.	2008	Caucasian	Weightlifting, skating	HIF1α Pro582Ser (rs11549465)	74	920
Ben Zaken et al.	2017	Caucasian	Swimming, running	IL6-174 G/C (rs1800795)	144	127
Ben Zaken et al.	2019	Caucasian	Weightlifting, running,	AGT Met235Thr (rs699)	125	86
Ben Zaken et al.	2020	Caucasian	Swimming, running	IL6-174 G/C (rs1800795)	110	64
Bosnyak et al.	2020	Caucasian	Wrestling, judo, boxing, karate, fencing,	HIF1α Pro582Ser (rs11549465)	38	90
Ciȩszczyk et al.	2011	Caucasian	Weightlifting, swimming, running,	HIF1ΑPro582Ser (rs11549465)	158	254
Ciȩszczyk et al.	2012	Caucasian	Weightlifting, swimming, running	AMPD1 C/T(rs17602729)	158	160
Drozdovska et al.	2013	Caucasian	Running, swimming, jump, throw	HIF1α Pro582Ser (rs11549465)	59	260
Eider et al.	2013	Caucasian	Weightlifting, swimming, running	IL6-174 G/C (rs1800795)	158	254
Eynon et al.	2010	Caucasian	Running	HIF1α Pro582Ser (rs11549465)	81	240
Eynon et al.	2011	Caucasian	Running	IL6-174 G/C (rs1800795)	81	205
Fedotovskaya et al.	2013	Caucasian	Weightlifting, skating, swimming, running, wrestling, boxing	AMPD1 C/T(rs17602729)	305	499
Gabbasov et al.	2013	Caucasian	Weightlifting, wrestling	HIF1α Pro582Ser (rs11549465)	208	1413
Ginevičiene et al.	2014	Caucasian	Running, throw, jump	AMPD1 C/T(rs17602729)	47	260
Gomez et al.	2009	Caucasian	Jump, throw, sprint	AGT Met235Thr (rs699)	63	119
Guilherma et al.	2018	Caucasian	Swimming, gymnastics, jump, throw, running, cycling, canoe, weightlifting, decathlon	AGT Met235Thr (rs699)	316	904
Miyamoto et al.	2017	Asian	Sprint, jump, throw, decathlon	AGT Met235Thr (rs699)	62	649
Peplonska et al.	2017	Caucasian	Sprint, swimming, skating, cycling	AGT Met235Thr (rs699)	188	451
Petr et al.	2022	Caucasian	Soccer	AMPD1 C/T(rs17602729)	99	107
Prancikeviciene et al.	2021	Caucasian	Running, jump, throw	AMPD1 C/T(rs17602729)	44	255
Ruiz et al.	2010	Caucasian	Jump, sprint	AGT Met235Thr (rs699)	53	100
Ruiz et al.	2010	Caucasian	Jump, sprint	IL6-174 G/C (rs1800795)	53	100
Zarebska et al.	2013	Caucasian	Running, powerlifting, weightlifting, throw, jump	AGT Met235Thr (rs699)	100	354

Table 2 shows the results of the frequency of *AGT* (rs699), *AMPD1* (rs17602729), *HIF1α* (rs11549465) and *IL-6* (rs1800795) single nucleotide polymorphisms of individuals forming the power athletes’ and the control group. Minimum three or more studies per SNP were included in the meta-analysis. When analyzing the overall allele frequency distributions, it was found that the frequency of the major alleles of *AGT* rs699 [OR: −0.25 [−0.42; −0.08]], *AMPD1* rs17602729 [OR: 0.45 [0.23; 0.68]], and *HIF1α* rs11549465 [OR: −0.55 [−0.79; −0.31]] polymorphisms were significantly lower in power athletes compared to the control group.

**Table 2:** Subgroup-analyses of the association between the genetic polymorphisms of the investigated genes and power athlete status.

Gene	Subgroup analysis	n	Major Allele	Wildtype	Allele based OR (95%Cl)	DM based OR (95%Cl)	RM based OR (95%Cl)
AGT Met235Thr (rs699)	Overall	7	Met	Met/Met	**−0.25(−0.42; −0.08)^R^ *p* = 0.004**	**−0.28(0.48; −0.08)^R^** ***p* = 0.005**	**0.28(0.11; 0.46)^F^** ***p* = 0.001**
	Caucasion	5	Met	Met/Met	**−0.33(−0.56; −0.10)^R^** ***p* = 0.005**	−0.22(−0.46; 0.01)^R^ *p* = 0.064	**0.55(0.30; 0.80)^F^** *p* = 0.001
AMPD1 C34T (rs17602729)	Overall	5	C	C/C	**0.45(0.23; 0.68)^F^** ***p* = 0.001**	**0.42(0.18; 0.67)^F^** ***p* = 0.001**	**−1.46(−2.59; −0.34)^R^** ***p* = 0.011**
	Male	3	C	C/C	0.22(−0.11; 0.55)^F^ *p* = 0.185	0.17(−0.20; 0.54)^F^ *p* = 0.361	−1.48(−3.18; 0.23)^R^ *p* = 0.090
	Elite	4	C	C/C	0.27(−0.03; 0.58)^F^ *p* = 0.080	0.27(−0.07; 0.61)^F^ *p* = 0.120	−1.02(−2.35; 0.31)^R^ *p* = 0.135
HIF1α (rs11549465)	Overall	6	Pro	Pro/Pro	−**0.55(−0.79; −0.31)^R^** ***p* = 0.001**	**−0.63(−0.93; −0.33)^R^** ***p* = 0.001**	−0.25(−0.70; 1.20)^R^ *p* = 0.606
	Elite	3	Pro	Pro/Pro	−0.36(−0.97; 0.24)^F^ *p* = 0.241	**−0.39(−0.78; 0.01)^F^** ***p* = 0.026**	0.42(−0.84; 1.68)^R^ *p* = 0.516
	Weightlifter	3	Pro	Pro/Pro	**−0.69(−0.97; −0.41)^R^** ***p* = 0.001**	**−0.82(−1.13; −0.52)^R^** ***p* = 0.001**	0.38(−1.43; 2.20)^R^ *p* = 0.677
IL6-174 G/C (rs1800795)	Overall	5	G	G/G	0.17(−0.02; 0.36) ^F^ *p* = 0.084	0.22(−0.02; 0.46) ^F^ *p* = 0.068	−0.09(−0.62; 0.44) ^R^ *p* = 0.746
	Male	3	G	G/G	**0.35(0.11; 0.58) ^F^** ***p* = 0.004**	**0.55(0.24; 0.86) ^F^** ***p* = 0.001**	0.11(−0.79; 1.01) ^R^ *p* = 0.810
	Swimming	3	G	G/G	−0.04(−0.35; 0.26) ^F^ *p* = 0.794	−0.14(−0.53; 0.25) ^F^ *p* = 0.478	−0.31(−1.01; 0.40) ^R^ *p* = 0.394
	Running	4	G	G/G	0.05(−0.23; 0.33) ^R^ *p* = 0.736	0.12(−0.30; 0.53) ^R^ *p* = 0.586	0.10(−0.82; 1.02) ^R^ *p* = 0.829
	Elite	4	G	G/G	−0.02(−0.40;0.36) ^R^ *p* = 0.917	−0.04(−0.33;0.26) ^F^ *p* = 0.797	0.08(−0.76;0.92) ^R^ *p* = 0.854

Abbreviation: OR, odds ratio; CI, confidence interval; DM, dominant; RM, recessive

When examining the genotype frequency distribution of *AGT* rs699 SNP, a significant difference was found in both the dominant model [OR: −0.28 [0.48; −0.08]] and the recessive model [OR: 0.28 [0.11; 0.46]] (*p* < 0.05). Similarly, significant differences were also found in the dominant [OR: 0.42 [0.18; 0.67]] and recessive models [OR: −1.46 [−2.59; −0.34]] of the *AMPD1* rs17602729 SNP genotype distribution (*p* < 0.05). In *HIF1α* rs11549465 SNP genotypes, only a significant difference was found in the dominant model [OR: −0.63 [−0.93; −0.33]] (*p* < 0.05). No significant difference was found in other overall allele and genotype frequency distributions (*p* > 0.05).

The subgroup analyses of allele frequency distributions and genotype frequency distributions of power athletes and control groups are shown in Table 2. The frequency distribution of the major allele (Met) of AGT rs699 SNP in Caucasian power athletes was found to be significantly lower than in the controls [OR: −0.33 [−0.56; −0.10]] (*p* < 0.05).

In the genotype frequency distributions of AGT rs699 in Caucasian power athletes, a significant difference was only found in the recessive model [OR: 0.55 [0.30; 0.80]] (*p* < 0.05). When examining the subgroup analysis of HIF1α rs11549465 SNP, it was found that the frequency distribution of the major allele (Pro) in weightlifters was statistically lower than in the controls [OR: −0.69 [−0.97; −0.41]] (*p* < 0.05). In terms of genotype frequency distributions, the dominant model was found to be significantly lower in elite power athletes [OR: −0.39 [−0.78; 0.01]] and weightlifters [OR: −0.82 [−1.13; −0.52]] (*p* < 0.05). It was determined that the frequency of the major allele (G) of IL-6 rs1800795 SNP in the subgroup of male power athletes was [OR: 0.35 [0.11; 0.58]] times higher and the frequency of the GG genotype was [OR: 0.55 [0.24; 0.86]] times higher than in the controls (*p* < 0.05). No other statistically significant differences were found in other analyses (*p* > 0.05).

**Figure 1:**
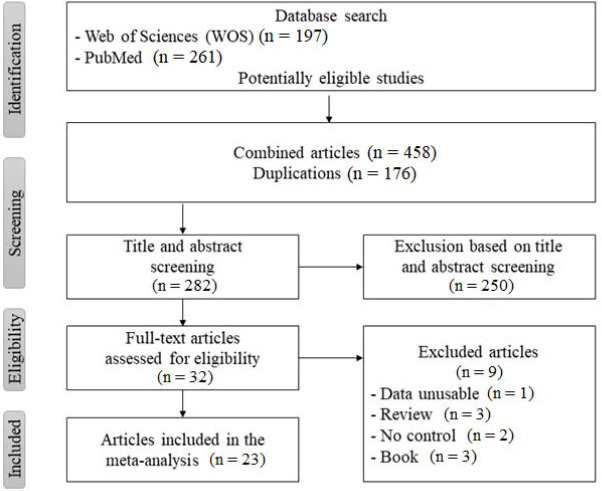
The flow diagram of included/excluded studies.

**Figure 2:**
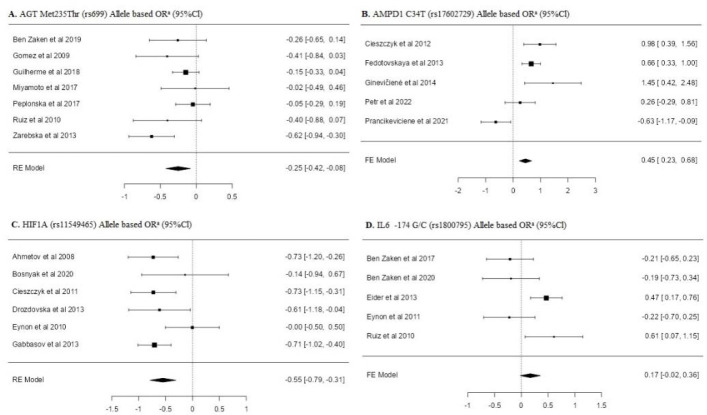
Allele based forest plots for AGT, AMPD1, HIF1α, IL-6 polymorphisms.

**Figure 3:**
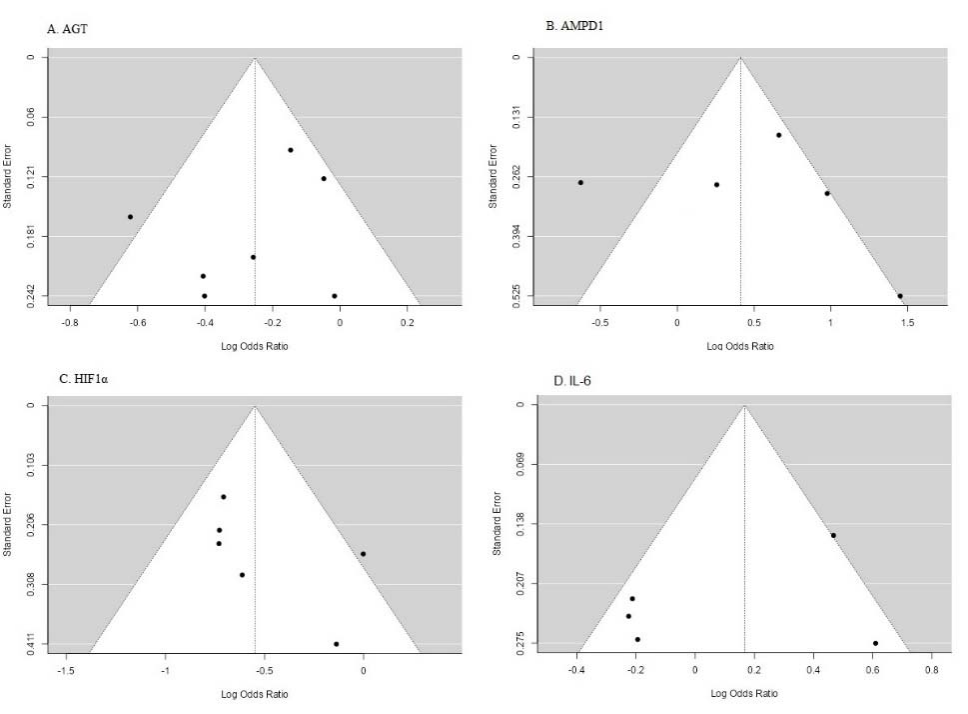
Allele based funnel plots for AGT, AMPD1, HIF1α, IL-6 polymorphisms.

## Discussion

The purpose of this study was to evaluate the genotype and allele frequency distributions of four popular genes that are believed to be associated with being a power athlete, using a meta-analysis approach. The data obtained from the genotype distributions were included in the analysis according to the ethnic background, gender, athlete’s performance level, and sport discipline. For this meta-analysis, a total of 23 articles were found to be suitable, and according to the results of the analysis, the distribution of *AGT* (rs 699), *AMPD1* (rs 17602729), *HIF1α* (rs 11549465), and *IL-6* (rs 1800795) polymorphisms in power athletes was evaluated.

In this study, we analyzed the distribution frequencies of the AGT (rs699) polymorphism in a total of 3570 individuals, consisting of 907 power athletes and 2663 control individuals. According to the results of the analysis, the Thr allele and the Thr/Thr genotype of the AGT rs699 polymorphism were more commonly observed in power athletes [OR = 0.28; 95%CI = 0.11; 0.46]. In the subgroup, the frequency of the Thr allele and the Thr/Thr genotype was found to be higher in power athletes of the Caucasian origin [OR = 0.55; 95%CI = 0.30; 0.80]. When the literature was examined, it was seen that there was only one meta-analysis study that examined the relationship between *AGT* and power athlete status. In a meta-analysis study conducted by Weyerstraß et al. (2018) which aimed to examine the genetic polymorphism distribution of power athletes, the *AGT* rs699 polymorphism distribution of a total of 216 power athletes of the Caucasian origin was analyzed. According to the results of that study, the distribution frequency of the Thr allele and the Thr/Thr genotype was significantly higher in power athletes compared to controls. This result is also consistent with our study. It is known that increased levels of angiotensin II enzyme seen in Thr carriers play a significant role in skeletal muscle growth (Pickering et al., 2019). Based on these findings, it can be said that the carriage of the Thr allele and the Thr/Thr genotype may be high in power athletes.

There are studies indicating that the frequency of the *AMPD1* rs17602729 C/C genotype is higher in power athletes (Fedotovskaya et al., 2013). In another study, male athletes with high anaerobic and short-term explosive power had a high rate of the C/C genotype (Ginevičiene et al., 2014). Similar studies have also observed that the frequency of the dominant model was higher in power athletes and could contribute to anaerobic performance (Peter Atanasov et al., 2015; Petr et al., 2022; Pranckeviciene et al., 2021). On the other hand, Cieszczyk et al. (2012) reported in a study which they carried out to examine the distribution of the *AMPD1* rs17602729 polymorphism in power athletes that the T allele was higher in the control group. In the literature, there is no meta-analysis examining the distribution of the *AMPD1* rs17602729 polymorphism in power athletes. According to our meta-analysis results, the frequency of the *AMPD1* (rs17602729) C allele [OR = 0.45; 95%CI = 0.23; 0.68] and the C/C genotype [OR = 0.42; 95%CI = 0.18; 0.67] in power athletes was found to be higher than in the control group. When sub-dimensions were examined, the *AMPD1* rs17602729 polymorphism distributions did not show any significant differences in any model, neither in men nor in elite power athletes. The results of this study help to look at the contradictory results found in the literature from a broader perspective.

Glycolysis is an important anaerobic energy source for power performance. One of the regulators of this metabolic pathway under low oxygen conditions is the transcription factor known as hypoxia-inducible factor 1α (MacIejewska-Skrendo et al., 2019). Particularly in sports that require speed and explosiveness, significant relationships have been observed showing higher frequencies of the *HIF1α* (rs11549465) Ser allele and the Ser/Ser genotype in power athletes. That meta-analysis included 3795 individuals of the Caucasian origin (power athletes = 618, control group = 3177) and analyzed their genetic polymorphism distributions of *HIF1α* (rs11549465). According to the results, the frequency of the Pro allele [OR = −0.55; 95%CI = −0.79; −0.31] and the Pro/Pro genotype [OR = −0.63; 95%CI = −0.93; −0.33] in power athletes was found to be less than in the control group. In the recessive model, no statistically significant difference was detected. When the distribution of the *HIF1α* rs11549465 polymorphism was evaluated according to elite power athlete status, the frequency distribution was lower in the dominant model compared to the control group. Moreover, the dominant model genotype distribution of weightlifters was even lower. When the literature is reviewed, there is no meta-analysis study examining the allele and genotype distribution of the *HIF1α* rs11549465 polymorphism in power athletes. A study by Ahmetov et al. (2008) found that the *HIF1α* (rs11549465) Ser allele was significantly higher in weightlifters and concluded that it increased the development of athletic performance. Similarly, some studies have also noted that the frequency of the *HIF1α* (rs11549465) Ser allele is higher in power athletes compared to control individuals (Ciȩszczyk et al., 2011; Drozdovska et al., 2013; Gabbasov et al., 2013). Studies by Eynon et al. (2010) and Bosnyák et al. (2020) support our findings. In their studies, no difference was found in the Ser/Ser genotype and Ser allele frequency distributions between endurance athletes, power athletes and control groups. However, more studies are needed to confirm these results.

Our study examined the distribution of genotype and allele frequencies of the *IL-6* rs1800795 polymorphism in relation to the athletes’ power status. The study found that only male power athletes had a significantly higher frequency of the G allele [OR = 0.35; 95%CI = 0.11; 0.58] and the G/G genotype [OR = 0.55; 95%CI = 0.24; 0.86] compared to controls. However, no significant relationship was found in other variables. The only meta-analysis on the *IL-6* rs1800795 polymorphism and gender variable by Weyerstraß et al. (2018) also found significant differences, thus supporting the findings of our research. Despite our study examining a total of 1296 subjects, including 546 power athletes and 750 controls, more sources are needed to investigate the relationship between *IL-6* rs1800795 polymorphism and power athlete status.

## Conclusions

In conclusion, the findings of this study provide valuable insights into the genetic factors associated with the athletes’ power status. The identified genetic polymorphisms, such as AGT rs699, AMPD1 rs17602729, HIF1α rs11549465, and IL-6 rs1800795, can be considered as potential markers for power athlete status. Specifically, the AGT rs699 Thr allele, HIF1α rs11549465 Ser allele and AMPD1 rs17602729 C allele were found to be more prevalent in power athletes. However, more research is needed to confirm these results and explore the relationships between genetic polymorphisms and the athletes’ power status. Pooling data from multiple studies through collaborative efforts and conducting larger-scale meta-analyses would provide a clearer understanding of the genetic factors determining the athletes’ power status.

## References

[ref1] Ahmetov, I. I., & Fedotovskaya, O. N. (2012). Sports genomics: Current state of knowledge and future directions. Cellular and Molecular Exercise Physiology, 1(1), 1–24. 10.7457/cmep.v1i1.e1

[ref2] Ahmetov, I. I., & Fedotovskaya, O. N. (2015). Current Progress in Sports Genomics. Advances in Clinical Chemistry, 70, 247–314. 10.1016/BS.ACC.2015.03.003 26231489

[ref3] Ahmetov, I. I., Hakimullina, A. M., Lyubaeva, E. v., Vinogradova, O. L., & Rogozkin, V. A. (2008). Effect of HIF1A gene polymorphism on human muscle performance. Bulletin of Experimental Biology and Medicine, 146(3), 351–353. 10.1007/S10517-008-0291-3 19240858

[ref4] Atanasov, P., Djarova, T., Kalinski, M., Petrov, L., Kaneva, R., Mugandani, S., Watson, G. & Jemni M. (2015). ACTN3 and AMPD1 Polymorphism and Genotype Combinations in Bulgarian Athletes Performing Wingate Test. Journal of Sports Science, 3(1), 1–10. 10.17265/2332-7839/2015.01.001

[ref5] Bosnyák, E., Trájer, E., Alszászi, G., Móra, Á., Györe, I., Udvardy, A., Tóth, M., & Szmodis, M. (2020). Lack of association between the GNB3 rs5443, HIF1A rs11549465 polymorphisms, physiological and functional characteristics. Annals of Human Genetics, 84(5), 393–399. 10.1111/ahg.12387 32391916

[ref6] Ciȩszczyk, P., Eider, J., Arczewska, A., Ostanek, M., Leońska-Duniec, A., Sawczyn, S., Ficek, K., Jascaniene, N., Kotarska, K., & Sygit, K. (2011). The HIF1A gene Pro582Ser polymorphism in Polish power-orientated athletes. Biology of Sport, 28(2), 111–114. 10.5604/945117

[ref7] Ciȩszczyk, P., Ostanek, M., Leońska-Duniec, A., Sawczuk, M., Maciejewska, A., Eider, J., Ficek, K., Sygit, K., & Kotarska, K. (2012). Distribution of the AMPD1 C34T polymorphism in Polish power-oriented athletes. Journal of Sports Sciences, 30(1), 31–35. 10.1080/02640414.2011.623710 22017426

[ref8] Corvol, P., & Jeunemaitre, X. (1997). Molecular genetics of human hypertension: role of angiotensinogen. Endocrine Reviews, 18(5), 662–677. 10.1210/edrv.18.5.0312 9331547

[ref9] Dias, R. G., Pereira, A. D. C., Negrão, C. E., & Krieger, J. E. (2007). Genetic polymorphisms determining of the physical performance in elite athletes. Revista Brasileira de Medicina Do Esporte, 13(3), 209–216. 10.1590/S1517-86922007000300016

[ref10] Drozdovska, S. B., Dosenko, V. E., Ahmetov, I. I., & Ilyin, V. N. (2013). The association of gene polymorphisms with athlete status in Ukrainians. Biology of Sport, 30(3), 163–167. 10.5604/20831862.1059168 24744483 PMC3944573

[ref11] Eynon, N., Alves, A. J., Meckel, Y., Yamin, C., Ayalon, M., Sagiv, M., & Sagiv, M. (2010). Is the interaction between HIF1A P582S and ACTN3 R577X determinant for power/sprint performance? Metabolism: Clinical and Experimental, 59(6), 861–865. 10.1016/J.METABOL.2009.10.003 20005538

[ref12] Eynon, N., Hanson, E. D., Lucia, A., Houweling, P. J., Garton, F., North, K. N., & Bishop, D. J. (2013). Genes for elite power and sprint performance: ACTN3 leads the way. Sports Medicine (Auckland, N.Z.), 43(9), 803–817. 10.1007/S40279-013-0059-4 23681449

[ref13] Faul, F., Erdfelder, E., Lang, A. G., & Buchner, A. (2007). G*Power 3: a flexible statistical power analysis program for the social, behavioral, and biomedical sciences. Behavior Research Methods, 39(2), 175–191. 10.3758/BF03193146 17695343

[ref14] Febbraio, M. A., & Pedersen, B. K. (2005). Contraction-induced myokine production and release: is skeletal muscle an endocrine organ? Exercise and Sport Sciences Reviews, 33(3), 114–119. 10.1097/00003677-200507000-00003 16006818

[ref15] Fedotovskaya, O. N., Danilova, A. A., & Akhmetov, I. I. (2013). Effect of AMPD1 gene polymorphism on muscle activity in humans. Bulletin of Experimental Biology and Medicine, 154(4), 489–491. 10.1007/S10517-013-1984-9/metrics 23486588

[ref16] Gabbasov, R. T., Arkhipova, A. A., Borisova, A. v., Hakimullina, A. M., Kuznetsova, A. v., Williams, A. G., Day, S. H., & Ahmetov, I. I. (2013). The HIF1A gene PRO582SER polymorphism in Russian strength athletes. Journal of Strength and Conditioning Research, 27(8), 2055–2058. 10.1519/jsc.0b013e31827f06ae 23222085

[ref17] Ginevičiene, V., Jakaitiene, A., Pranculis, A., Milašius, K., Tubelis, L., & Utkus, A. (2014). AMPD1 rs17602729 is associated with physical performance of sprint and power in elite Lithuanian athletes. BMC Genetics, 15(1), 1–9. 10.1186/1471-2156-15-58/TABLES/3 24885427 PMC4032451

[ref18] Gomez-Gallego, F., Santiago, C., González-Freire, M., Yvert, T., Muniesa, C. A., Serratosa, L., Altmäe, S., Ruiz, J. R., & Lucia, A. (2009). The C allele of the AGT Met235Thr polymorphism is associated with power sports performance. Applied Physiology, Nutrition, and Metabolism, 34(6), 1108–1111. 10.1139/H09-108 20029521

[ref19] Ipekoglu, G., Bulbul, A., & Cakir, H. I. (2022). A meta-analysis on the association of ACE and PPARA gene variants and endurance athletic status. Journal of Sports Medicine and Physical Fitness, 62(6), 795–802. 10.23736/S0022-4707.21.12417-X 34028240

[ref20] Maciejewska-Skrendo, A., Mieszkowski, J., Kochanowicz, A., Niespodziński, B., Cieszczyk, P., Leźnicka, K., Leońska-Duniec, A., Kolbowicz, M., Kaczmarczyk, M., Piskorska, E., Stankiewicz, B., Stępniak, R., Mostowik, A., Zawartka, M., Rzeszutko-Bełzowska, A., Massidda, M., Caló, C. M., Kemerytė-Riaubienė, E., & Sawczuk, M. (2021). Does the PPARA Intron 7 Gene Variant (rs4253778) Influence Performance in Power/Strength-Oriented Athletes? A Case-Control Replication Study in three Cohorts of European Gymnasts. Journal of Human Kinetics, 79, 77–85. 10.2478/hukin-2020-0060 34400988 PMC8336554

[ref21] Maciejewska-Skrendo, A., Ciȩszczyk, P., Chycki, J., Sawczuk, M., & Smółka, W. (2019). Genetic Markers Associated with Power Athlete Status. Journal of Human Kinetics, 68(1), 17–36. 10.2478/hukin-2019-0053 31531130 PMC6724599

[ref22] Petersen, A. M., Pedersen, B. K. (2005). The anti-inflammatory effect of exercise. Journal of Applied Physiology, 98(4), 1154–62. 10.1152/japplphysiol.00164.2004 15772055

[ref23] Petr, M., Thiel, D., Kateřina, K., Brož, P., Malý, T., Zahálka, F., Vostatková, P., Wilk, M., Chycki, J., & Stastny, P. (2022). Speed and power-related gene polymorphisms associated with playing position in elite soccer players. Biology of Sport, 39(2), 355–366. 10.5114/biolsport.2022.105333 35309536 PMC8919892

[ref24] Pickering, C., Suraci, B., Semenova, E. A., Boulygina, E. A., Kostryukova, E. S., Kulemin, N. A., Borisov, O. v., Khabibova, S. A., Larin, A. K., Pavlenko, A. v., Lyubaeva, E. v., Popov, D. v., Lysenko, E. A., Vepkhvadze, T. F., Lednev, E. M., Leonska-Duniec, A., Pajak, B., Chycki, J., Moska, W., … Ahmetov, I. I. (2019). A Genome-Wide Association Study of Sprint Performance in Elite Youth Football Players. Journal of Strength and Conditioning Research, 33(9), 2344–2351. 10.1519/jsc.0000000000003259 31343553

[ref25] Pitsiladis, Y., Wang, G., Wolfarth, B., Scott, R., Fuku, N., Mikami, E., He, Z., Fiuza-Luces, C., Eynon, N., & Lucia, A. (2013). Genomics of elite sporting performance: what little we know and necessary advances. British Journal of Sports Medicine, 47(9), 550–555. 10.1136/bjsports-2013-092400 23632745

[ref26] Pranckeviciene, E., Gineviciene, V., Jakaitiene, A., Januska, L., & Utkus, A. (2021). Total Genotype Score Modelling of Polygenic Endurance-Power Profiles in Lithuanian Elite Athletes. Genes, 12(7), 1–18. 10.3390/genes12071067 PMC8306147 34356082

[ref27] Rauramaa, R., Kuhanen, R., Lakka, T. A., Väisänen, S. B., Halonen, P., Alén, M., Rankinen, T., & Bouchard, C. (2002). Physical exercise and blood pressure with reference to the angiotensinogen M235T polymorphism. Physiological Genomics, 10(2), 71–77. 10.1152/physiolgenomics.00050.2002 12181364

[ref28] Rubio, J. C., Martín, M. A., Rabadán, M., Gómez-Gallego, F., San Juan, A. F., Alonso, J. M., Chicharro, J. L., Pérez, M., Arenas, J., & Lucia, A. (2005). Frequency of the C34T mutation of the AMPD1 gene in worldclass endurance athletes: Does this mutation impair performance? Journal of Applied Physiology, 98(6), 2108–2112. 10.1152/japplphysiol.01371.2004/asset/images/large/zdg0060538460001.jpeg 15677729

[ref29] Ruiz, J. R., Arteta, D., Buxens, A., Artieda, M., Gómez-Gallego, F., Santiago, C., Yvert, T., Moran, M., & Lucia, A. (2010). Can we identify a power-oriented polygenic profile? Journal of Applied Physiology, 108(3), 561–566. 10.1152/japplphysiol.01242.2009/asset/images/large/zdg0031089350003.jpeg 20044471

[ref30] Serrano, A. L., Baeza-Raja, B., Perdiguero, E., Jardí, M., & Muñoz-Cánoves, P. (2008). Interleukin-6 is an essential regulator of satellite cell-mediated skeletal muscle hypertrophy. Cell Metabolism, 7(1), 33–44. 10.1016/j.cmet.2007.11.011 18177723

[ref31] Tanimoto, K., Yoshiga, K., Eguchi, H., Kaneyasu, M., Ukon, K., Kumazaki, T., Oue, N., Yasui, W., Imai, K., Nakachi, K., Poellinger, L., & Nishiyama, M. (2003). Hypoxia-inducible factor-1alpha polymorphisms associated with enhanced transactivation capacity, implying clinical significance. Carcinogenesis, 24(11), 1779–1783. 10.1093/carcin/bgg132 12919954

[ref32] Weyerstraß, J., Stewart, K., Wesselius, A., & Zeegers, M. (2018). Nine genetic polymorphisms associated with power athlete status-A Meta-Analysis. Journal of Science and Medicine in Sport, 21(2), 213–220. 10.1016/j.jsams.2017.06.012 28666769

[ref33] Zarebska, A., Sawczyn, S., Kaczmarczyk, M., Ficek, K., Maciejewska-Karowska, A., Sawczuk, M., Leonska-Duniec, A., Eider, J., Grenda, A., & Cieszczyk, P. (2013). Association of rs699 (m235t) polymorphism in the agt gene with power but not endurance athlete status. Journal of Strength and Conditioning Research, 27(10), 2898–2903. 10.1519/jsc.0b013e31828155b5 23287839

